# Reverse Takotsubo Cardiomyopathy Triggered by Undiagnosed Right Adrenal Pheochromocytoma: A Rare Occurrence

**DOI:** 10.7759/cureus.40924

**Published:** 2023-06-25

**Authors:** Hamad Ahmad, Hoore Jannat, Urooj Khan, Noaman Ahmad

**Affiliations:** 1 Internal Medicine, Westchester Medical Center, New York, USA; 2 Internal Medicine, Khyber Medical College, Peshawar, PAK; 3 Internal Medicine, Khyber Medical University, Peshawar, PAK; 4 Internal Medicine, Huntsville Hospital, Huntsville, USA

**Keywords:** adrenalectomy, inotropic support, adrenal pheochromocytoma, cardiogenic shock, reverse takotsubo cardiomyopathy

## Abstract

Takotsubo cardiomyopathy (TTC), also known as stress cardiomyopathy or broken heart syndrome, is a condition characterized by transient left ventricular dysfunction resembling myocardial infarction but without obstructive coronary artery disease. We present a rare case of a 59-year-old patient with cardiogenic shock (CS) caused by reverse TTC triggered by an undiagnosed right adrenal pheochromocytoma tumor. The patient initially presented with chronic headaches and difficulty breathing, and their condition rapidly deteriorated, necessitating intubation and inotropic support. Diagnostic tests confirmed the diagnosis of reverse TTC, and further investigation revealed an actively growing adrenal mass suggestive of a pheochromocytoma. The patient responded well to treatments, including the use of intra-aortic balloon pump support and subsequent weaning. A right adrenalectomy confirmed the presence of a pheochromocytoma. This case highlights the association between pheochromocytoma and reverse TTC, emphasizing the need to consider this rare etiology in patients presenting with CS. Long-term monitoring is crucial due to the risk of recurrence, even after tumor removal.

## Introduction

Takotsubo cardiomyopathy (TTC), also called stress cardiomyopathy and broken heart syndrome, is a syndrome characterized by transient regional systolic dysfunction, principally of the left ventricle (LV), mimicking myocardial infarction (MI), but in the absence of angiographic evidence of obstructive coronary artery disease or acute plaque rupture [1]. In most cases of stress cardiomyopathy, the regional wall motion abnormality extends beyond the territory perfused by a single epicardial coronary artery. Different patterns of left ventricular dysfunction have been reported, with the most common being the apical type, causing apical ballooning; other less common types include the midventricular type, the basal type (reverse TTC), and the global type [2]. The pathogenesis of TTC is not well understood, although its association with physical or emotional stress suggests that this disorder may be caused by diffuse catecholamine-induced microvascular spasm or dysfunction, resulting in myocardial stunning, or by direct catecholamine-associated myocardial toxicity [3]. Rarely, a pheochromocytoma, which is a catecholamine-secreting tumor that arises from chromaffin cells of the adrenal medulla and the sympathetic ganglia, can lead to TTC and, very rarely, can be its first presentation [4]. Here, we present a rare and interesting case of a patient who presented with cardiogenic shock (CS) secondary to reverse TTC triggered by an undiagnosed right adrenal pheochromocytoma tumor.

## Case presentation

**Figure 1:**
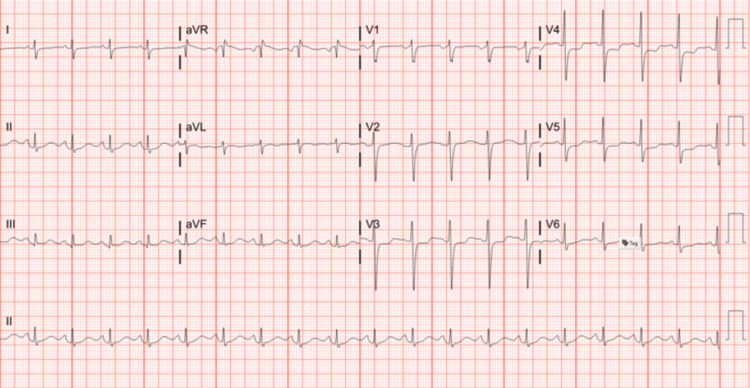
EKG: sinus tachycardia with probable left atrial enlargement and borderline right axis deviation EKG: electrocardiogram

A 59-year-old patient with a medical history of hypertension and diverticulitis post-partial colectomy presented with worsening chronic headaches and new-onset difficulty breathing. On arrival, the patient was drowsy, tachycardiac, tachypneic, hypertensive with blood pressure ranging from 180/100 to 190/110, and hypoxic on room air, necessitating the use of supplemental oxygen. On examination, the patient had reduced breath sounds at the lung bases bilaterally. Shortly after admission, the patient's respiratory and mental status deteriorated significantly, requiring endotracheal intubation for respiratory support and for protection of the airway. Laboratory tests revealed an elevated white blood cell count of 25000 k/mm^3^ (normal 4-11), an elevated creatinine of 1.58 mg/dl (normal 0.06-1.2), elevated liver enzymes of aspartate aminotransferase (AST) 160 U/L (normal 5-35), alanine transaminase (ALT) 308 U/L (normal 15-60), and alkaline phosphatase (ALP) 307 U/L (normal 60-120), and rapidly rising troponin 0.04-->1.6-->14-->25 (normal < 0.02). An electrocardiogram (EKG) revealed a sinus tachycardia with probable left atrial enlargement and borderline right axis deviation with no ST segment changes (Figure 1).

**Figure 2:**
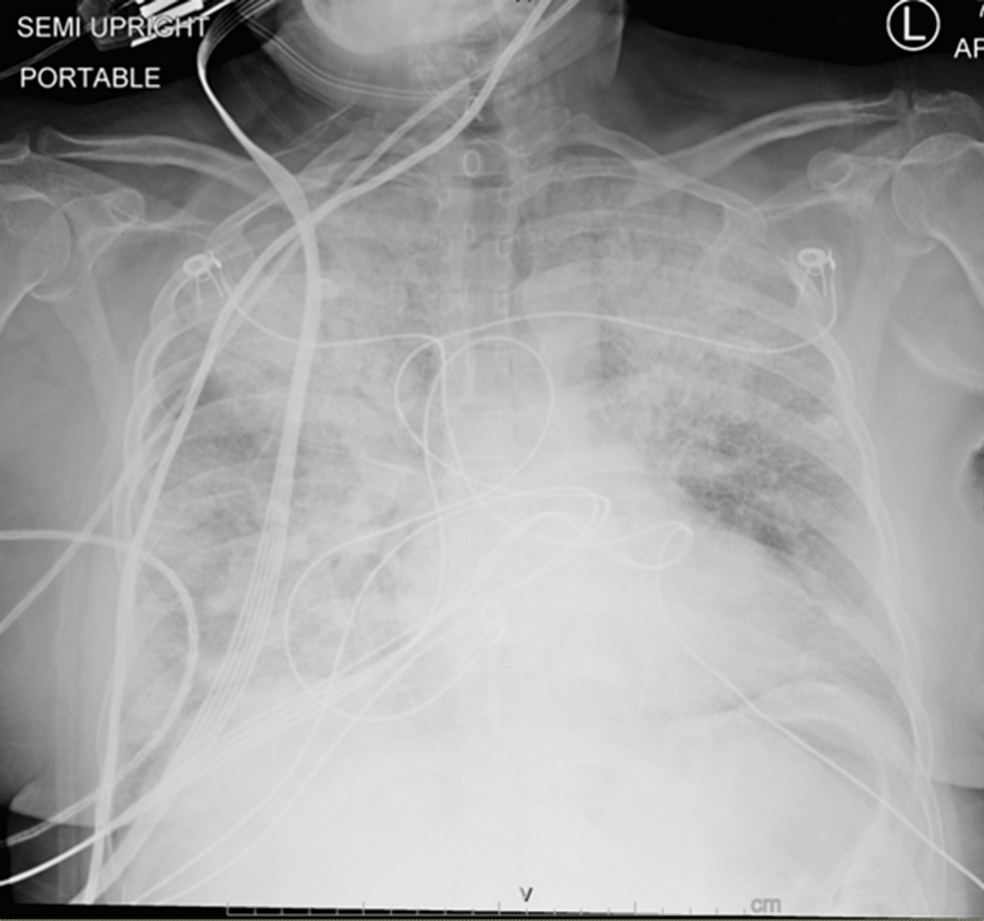
Chest X-ray: bilateral interstitial opacities

A chest X-ray showed bilateral interstitial hazy opacities (Figure 2).

**Figure 3:**
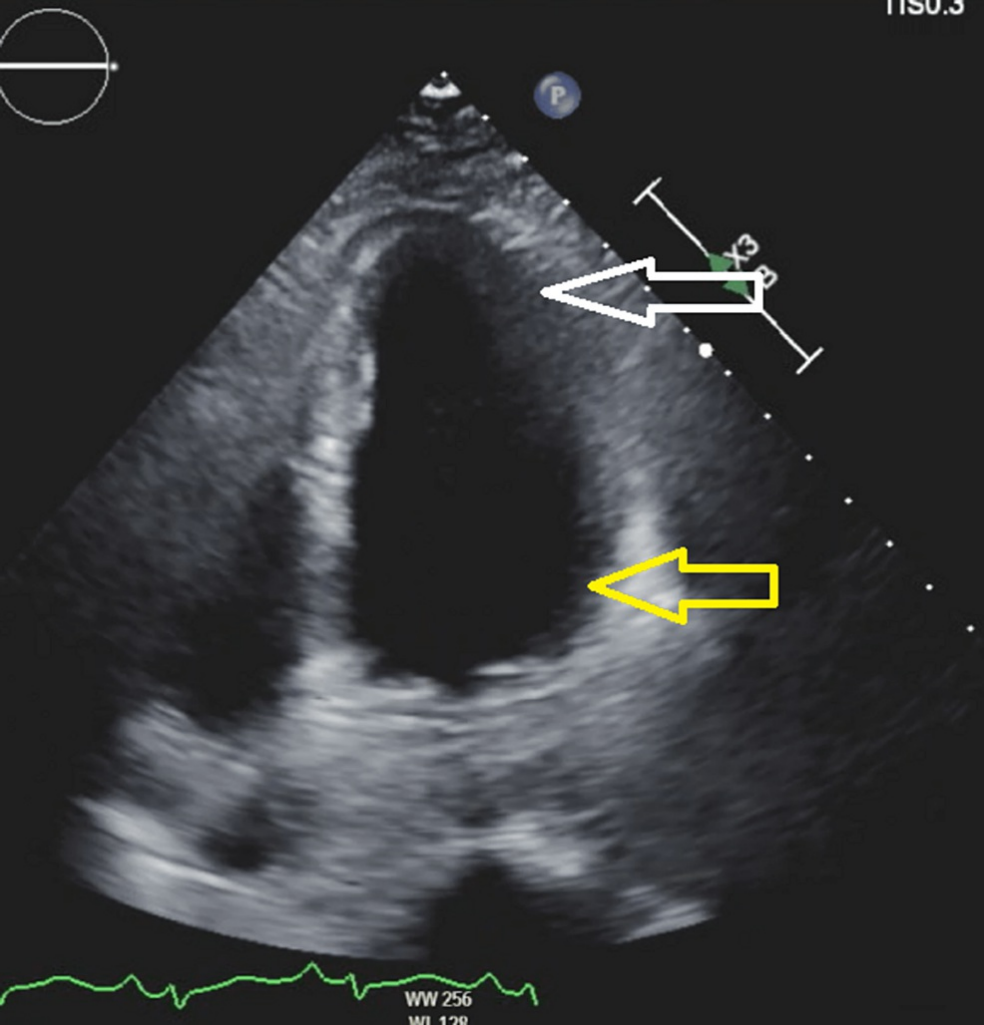
TTE (four-chamber view): apex (white arrow) and base (yellow arrow) of the LV chamber in diastolic relaxation TTE: transthoracic echocardiogram; LV: left ventricle

**Figure 4:**
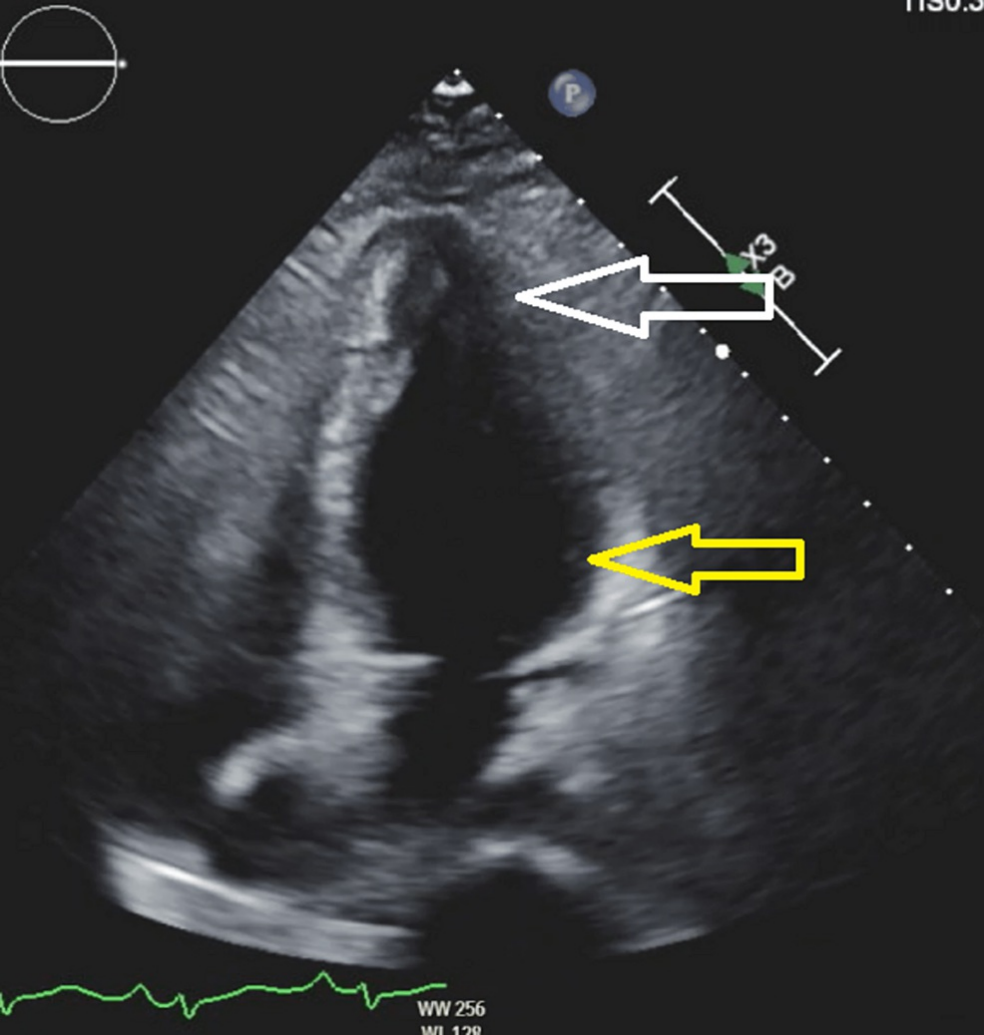
TTE: hyperkinetic apex (white arrow) and hypokinetic base (yellow arrow) of the LV chamber in systole TTE: transthoracic echocardiogram; LV: left ventricle

Transthoracic echocardiography (TTE) showed a mildly to moderately reduced ejection fraction, increased contractility at the apex, and decreased contractility at the base of the heart (Figures 3-4).

The patient's hemodynamic status decompensated acutely secondary to CS, as evident by severe hypotension (blood pressure of 70/40 mmHg) with associated worsening liver function and kidney function, necessitating the use of inotropic support (norepinephrine and dopamine). The patient was taken to the catherization suite for emergency left- and right-heart catheterization and later transferred to the coronary care unit for closer monitoring. Left-heart catheterization showed normal coronary arteries with no evidence of coronary artery disease or acute plaque processes. Right-heart catheterization showed significantly elevated filling pressures. An intra-aortic balloon pump (IABP) was inserted to provide cardiac support. Considering the absence of coronary artery disease, elevated troponin levels, and the low ejection fraction with the hypokinetic base and hyperkinetic apex on TTE, reverse TTC was the most likely cause of the CS. Other common secondary causes of cardiomyopathy, including HIV and viral infections, thyroid-related conditions, hemochromatosis, alcoholism, and toxic metabolism, were ruled out.

**Figure 5:**
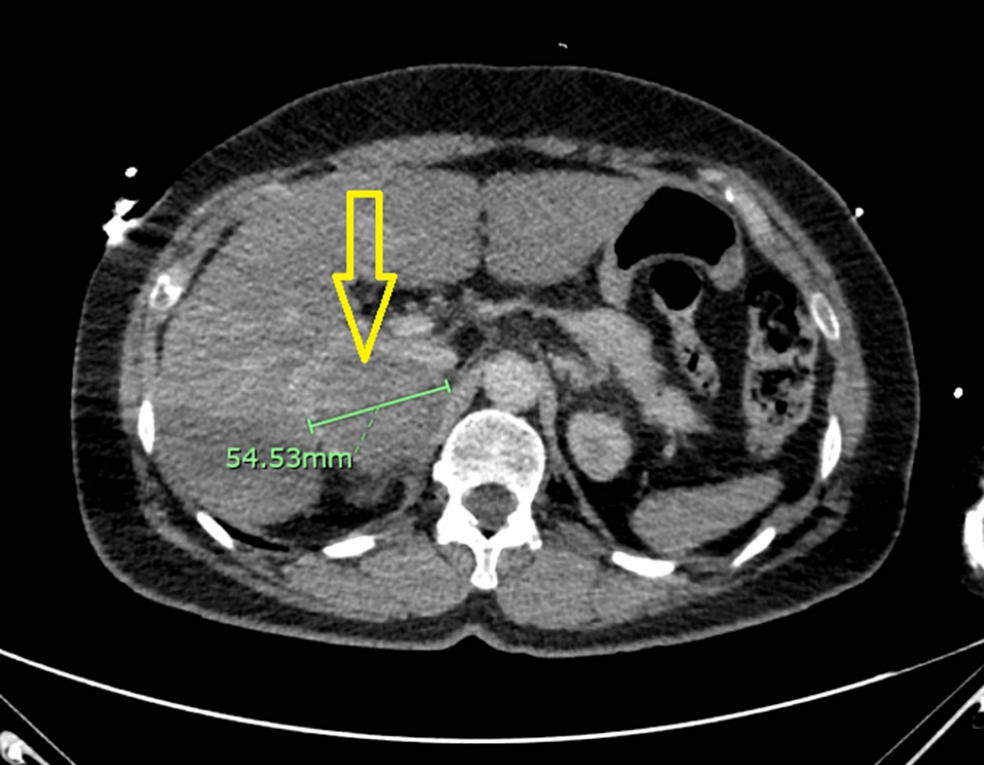
CT scan abdomen: showed 5.4 cm mass (yellow arrow) in the right adrenal gland highly suspicious for pheochromocytoma CT: computed tomography

Upon obtaining a detailed medical history, it was discovered that the patient was diagnosed with a right adrenal mass five years ago but did not undergo further evaluation. A subsequent abdominal computed tomography (CT) scan confirmed the presence of a right adrenal mass, which had nearly doubled in size since the initial diagnosis (Figure 5).

Given the patient's history of hypertension, headache, abdominal pain, and tachycardia upon presentation and subsequent CS from reverse TTC, the actively growing adrenal mass is likely a pheochromocytoma. Further investigations revealed elevated levels of metanephrines and catecholamines in both urine and plasma samples. On the third day of hospitalization, the patient's cardiovascular and respiratory status improved, and the patient was successfully weaned off the mechanical ventilator. The IABP was discontinued the following day. The patient did not need goal-directed medical therapy (GDMT) as the repeat TTE indicated normalization of the cardiac function with an ejection fraction of 55%, with resolution of hyperkinesia at the apex and hypokinesia at the base of the heart. To obtain better visualization of the adrenal mass, the patient underwent magnetic resonance imaging (MRI), which revealed slight enlargement and internal bleeding within the 6-cm right adrenal gland mass, suggesting the possibility of a benign or malignant neoplasm, including pheochromocytoma.

**Figure 6:**
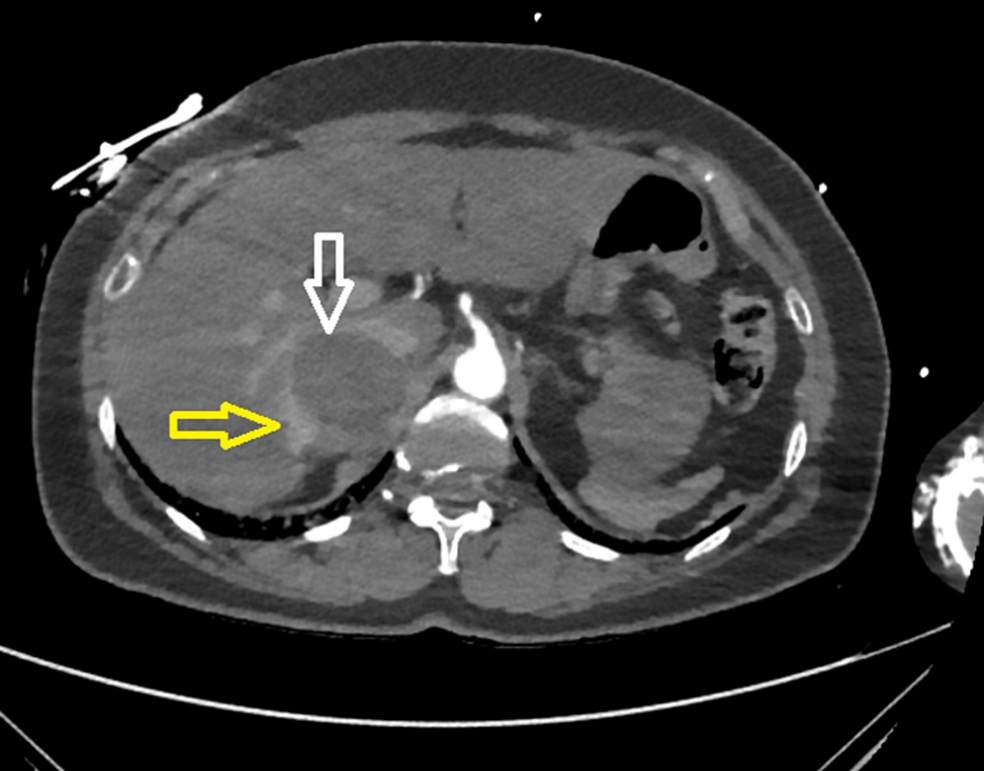
MRI abdomen showing a 5.5 cm mass (white arrow) and an internal bleed (yellow arrow) in the right adrenal gland MRI: magnetic resonance imaging

Given the clinical presentation consistent with a pheochromocytoma crisis in the context of adrenal hemorrhage, the patient underwent a right adrenalectomy. The subsequent biopsy confirmed the diagnosis of a pheochromocytoma measuring 4.8 cm in its largest dimension and exhibiting hemorrhage, central necrosis, and cystic degeneration. The patient was discharged with a plan for surveillance CT of the abdomen and pelvis in one year to evaluate for recurrence. The patient remained asymptomatic on the one-month follow-up and had normal serum metanephrines on repeat testing.

## Discussion

TTC was first described in 1990 in Japan and has since been increasingly recognized around the world [1]. TTC occurs in approximately 1-2% of patients presenting with troponin-positive suspected acute coronary syndrome or suspected ST-elevation MI [5]. The pathogenesis of TTC is not well understood, although it is postulated that catecholamines play a key role, causing microvascular spasm or dysfunction with resultant myocardial stunning or direct catecholamine-associated myocardial toxicity [3]. Hence, TTC is commonly seen among individuals exposed to physical or emotional stress. A prospective study of 92 patients admitted to a medical intensive care unit with a noncardiac diagnosis and no prior history of cardiac disease found that 26 patients (28%) had LV apical ballooning consistent with TTC [6]. Around 5% of patients with TTC develop CS, which has a high mortality rate [2]. CS is generally treated with ionotropic agents, such as norepinephrine, dopamine, and/or dobutamine. In the case of TTC, it becomes challenging due to the ambivalent role of catecholamines. These agents help maintain organ perfusion, but on the other hand, they lead to deterioration of the cardiomyopathy by further exacerbating the catecholamine surge [7]. Mechanical cardiac support devices, such as the IABP, Impella CP® (Abiomed, USA), and veno-arterial extracorporeal membrane oxygenation (va-ECMO), prove to be very beneficial in these situations as they provide hemodynamic stability, resulting in adequate organ perfusion, and also reduce catecholamine dependency and break the TTC vicious cycle [8]. Hence, our patient was initially started on ionotropic support for CS, but as soon as TTC was suspected, the IABP was initiated, and the patient was weaned off the ionotropic support to reduce the catecholamine surge, which is the driving force, resulting in further LV dysfunction. As our patient responded well, further escalation of mechanical support was not done, and the patient was weaned off the IABP as the LV recovered.

In most cases, catecholamine surges result from physical or emotional stress, but in rare cases, an incidental pheochromocytoma can trigger the surge. Our patient, though, had a known history of adrenal mass but did not have any workup done as the patient was mostly asymptomatic besides intermittent headaches. The classic triad for pheochromocytoma consists of episodic headache, sweating, and tachycardia [1]. Approximately one-half have paroxysmal hypertension; most of the rest have either primary hypertension (formerly called "essential" hypertension) or normal blood pressure [9]. The diagnosis of pheochromocytoma is typically made by measurements of urinary and plasma fractionated metanephrines and catecholamines. Our patient had elevated plasma and urinary metanephrine and normetanephrine levels for more than 24 hours after stopping inotropes to avoid interference by the vasopressors. As in the case of any endocrine pathology, biochemical confirmation of the diagnosis should be followed by radiological evaluation [10]. Our patient had a repeat CT scan, which showed a significant increase in size compared to the previous scan five years ago. A minimally invasive approach to the adrenal gland is the procedure of choice for patients with small, solitary intraadrenal pheochromocytomas that have no malignant radiologic features [11]. Complications of surgery for pheochromocytoma are primarily due to severe preoperative hypertension, high-secretion tumors, or repeat intervention for recurrence [12]. Surgical removal of a pheochromocytoma does not always lead to long-term cure of pheochromocytoma or hypertension, even in patients with a benign tumor, due to the risk of recurrence, more so in familial cases compared to sporadic ones (16% vs. 3%) [13-14]. Hence, long-term monitoring should be done in all patients, even those apparently cured, as recurrences may occur years later.

## Conclusions

This case report highlights reverse TTC triggered by an undiagnosed adrenal pheochromocytoma tumor. Prompt recognition and management are crucial, including the use of mechanical support devices. Consideration of rare etiologies, such as pheochromocytoma, is important in TTC. Long-term monitoring is necessary due to the risk of recurrence even after surgical removal. This report emphasizes the need for comprehensive evaluation and follow-up in patients with TTC and the interplay between catecholamines, adrenal tumors, and cardiac dysfunction.

## References

[1] Singh T, Khan H, Gamble DT, Scally C, Newby DE, Dawson D (2022). Takotsubo syndrome: pathophysiology, emerging concepts, and clinical implications. Circulation.

[2] Templin C, Ghadri JR, Diekmann J (2015). Clinical features and outcomes of takotsubo (stress) cardiomyopathy. N Engl J Med.

[3] Nef HM, Möllmann H, Kostin S (2007). Tako-Tsubo cardiomyopathy: intraindividual structural analysis in the acute phase and after functional recovery. Eur Heart J.

[4] Kassim TA, Clarke DD, Mai VQ, Clyde PW, Mohamed Shakir KM (2008). Catecholamine-induced cardiomyopathy. Endocr Pract.

[5] Prasad A, Dangas G, Srinivasan M, Yu J, Gersh BJ, Mehran R, Stone GW (2014). Incidence and angiographic characteristics of patients with apical ballooning syndrome (takotsubo/stress cardiomyopathy) in the HORIZONS-AMI trial: an analysis from a multicenter, international study of ST-elevation myocardial infarction. Catheter Cardiovasc Interv.

[6] Park JH, Kang SJ, Song JK, Kim HK, Lim CM, Kang DH, Koh Y (2005). Left ventricular apical ballooning due to severe physical stress in patients admitted to the medical ICU. Chest.

[7] Coupez E, Eschalier R, Pereira B (2014). A single pathophysiological pathway in Takotsubo cardiomyopathy: Catecholaminergic stress. Arch Cardiovasc Dis.

[8] Mariani S, Richter J, Pappalardo F (2020). Mechanical circulatory support for Takotsubo syndrome: a systematic review and meta-analysis. Int J Cardiol.

[9] Baguet JP, Hammer L, Mazzuco TL, Chabre O, Mallion JM, Sturm N, Chaffanjon P (2004). Circumstances of discovery of phaeochromocytoma: a retrospective study of 41 consecutive patients. Eur J Endocrinol.

[10] Stein PP, Black HR (1991). A simplified diagnostic approach to pheochromocytoma. A review of the literature and report of one institution's experience. Medicine (Baltimore).

[11] Aliyev S, Karabulut K, Agcaoglu O, Wolf K, Mitchell J, Siperstein A, Berber E (2013). Robotic versus laparoscopic adrenalectomy for pheochromocytoma. Ann Surg Oncol.

[12] Plouin PF, Duclos JM, Soppelsa F, Boublil G, Chatellier G (2001). Factors associated with perioperative morbidity and mortality in patients with pheochromocytoma: analysis of 165 operations at a single center. J Clin Endocrinol Metab.

[13] Amar L, Servais A, Gimenez-Roqueplo AP, Zinzindohoue F, Chatellier G, Plouin PF (2005). Year of diagnosis, features at presentation, and risk of recurrence in patients with pheochromocytoma or secreting paraganglioma. J Clin Endocrinol Metab.

[14] Holscher I, van den Berg TJ, Dreijerink KM, Engelsman AF, Nieveen van Dijkum EJ (2021). Recurrence rate of sporadic pheochromocytomas after curative adrenalectomy: a systematic review and meta-analysis. J Clin Endocrinol Metab.

